# Deep Monocular Depth Estimation Based on Content and Contextual Features

**DOI:** 10.3390/s23062919

**Published:** 2023-03-08

**Authors:** Saddam Abdulwahab, Hatem A. Rashwan, Najwa Sharaf, Saif Khalid, Domenec Puig

**Affiliations:** Department of Computer Engineering and Mathematics, Universitat Rovira i Virgil, Campus Sescelades, Avinguda dels Paisos Catalans, 26, 43007 Tarragona, Spain

**Keywords:** deep learning, monocular depth estimation, autoencoder network, contextual semantic information

## Abstract

Recently, significant progress has been achieved in developing deep learning-based approaches for estimating depth maps from monocular images. However, many existing methods rely on content and structure information extracted from RGB photographs, which often results in inaccurate depth estimation, particularly for regions with low texture or occlusions. To overcome these limitations, we propose a novel method that exploits contextual semantic information to predict precise depth maps from monocular images. Our approach leverages a deep autoencoder network incorporating high-quality semantic features from the state-of-the-art HRNet-v2 semantic segmentation model. By feeding the autoencoder network with these features, our method can effectively preserve the discontinuities of the depth images and enhance monocular depth estimation. Specifically, we exploit the semantic features related to the localization and boundaries of the objects in the image to improve the accuracy and robustness of the depth estimation. To validate the effectiveness of our approach, we tested our model on two publicly available datasets, NYU Depth v2 and SUN RGB-D. Our method outperformed several state-of-the-art monocular depth estimation techniques, achieving an accuracy of 85%, while minimizing the error Rel by 0.12, RMS by 0.523, and $log_{10}$ by 0.0527. Our approach also demonstrated exceptional performance in preserving object boundaries and faithfully detecting small object structures in the scene.

## 1. Introduction

Depth estimation is one of the most important computer vision tasks due to its use in face recognition, video surveillance, and indoor and outdoor robot navigation. Estimating depth maps from monocular images is based on inferring 3D forms and comprehending high-level scene structures. However, due to the difficulties of extracting information from a single image, such as changes in geometry, scene texture, occlusion of scene borders, and ambiguity, using a single image for predicting depth maps is challenging for several reasons [1]. As a result, the boundaries of the objects become blurry, which lowers the accuracy of the estimated depth maps.

Computer vision tasks, such as monocular depth estimation, have significantly boosted performance due to deep neural networks. Deep neural networks also significantly improve semantic segmentation techniques. Thus, by localizing the objects and detecting their boundaries, monocular depth estimation can considerably benefit from semantic data to estimate depth more precisely. As a result, focusing on contextual information in input images may be advantageous for accurate monocular depth estimation.

**Figure 1:**
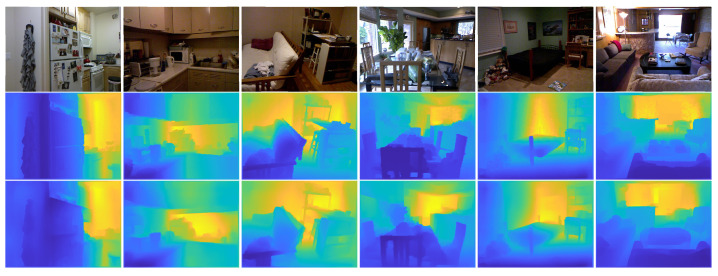
Comparison of estimated depth maps with our model with the NYU Depth-v2 dataset: (Row 1) Input images, (Row 2) ground-truth depth images, and (Row 3) resulting depth images.

Figure 1 shows the comparison of estimated depth maps with our model with the NYU Depth-v2 dataset.

In our previous work, such as [2,3], we have depended on the content and structure features extracted by an autoencoder for depth estimation. However, in this paper, we aim to merge features extracted from depth information and ones extracted from semantic context information to preserve the object’s boundaries. Thus, we suggest using two autoencoder networks in this work, each with an encoder and decoder. In order to extract high-level content, context and structure features from the input images, the first encoder network is trained from scratch. To preserve the discontinuities of the objects, we add contextual semantic features to the high-level features extracted by the first encoder using a pre-trained encoder network of the semantic segmentation model introduced in [4]. The extracted contents and contextual semantic features will be concatenated and fed into the decoder network to create the depth map and preserve object discontinuities. The following are the main contributions of this work:

- This work proposes a deep autoencoder network that leverages the benefits of squeeze-and-excitation networks (SENets) presented in [5]. SENets use the convolutional neural network (CNN) blocks to enhance channel interdependencies and improve feature representation without significant computational overhead. The proposed network is designed to extract precise content and structural information from monocular images, leveraging the power of deep learning to accurately predict depth from RGB input.
- This work proposes to enhance the accuracy of depth prediction for monocular images by leveraging the well-known semantic segmentation model HRNet-V2, as presented in [6]. HRNet-V2 enriches the content features with contextual semantic information, enabling the model to capture object boundaries better and maintain high-level representations of small objects in images. By integrating the strengths of HRNet-V2 with a deep learning approach to monocular depth prediction, this study aims to advance the state-of-the-art technologies in this field.
- The proposed model is an integrated framework combining two autoencoders to accurately predict high-resolution depth maps from monocular images. By leveraging the strengths of both models, the integrated framework is designed to provide a more robust and accurate prediction of depth, even in challenging scenarios. The proposed framework aims to advance the field of monocular depth prediction by providing a unified approach that can capture the richness and complexity of the real world while maintaining computational efficiency.

**Figure 2:**
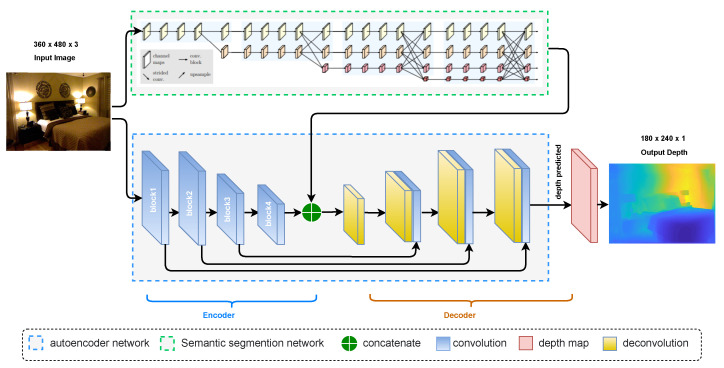
General overview of the proposed depth estimation model.

Figure 2 shows the proposed monocular depth estimation.

The rest of the article is structured as follows. The related work is summarized in Section 2. The proposed methodology for monocular depth estimation is described in Section 3. The experimental findings and performance are shown in Section 4. Section 5 concludes this work and suggests additional research directions.

## 2. Related Work

One of the key objectives of computer vision is to estimate the depth map from a monocular, stereo, or multi-view images. We concentrate on monocular depth estimation in this paper. The ability to predict depth images from a single image has received much attention over the years and has been approached from various angles. Here, we focus on the achievements of recent years.

In [7], the authors presented a method for estimating depth maps from a single RGB image using a multi-scale deep convolutional neural network (CNN). The proposed method is based on the idea that an image’s geometric and photometric constraints can be used to infer depth. The authors use a CNN to extract features from the image at multiple scales to achieve this. These features are then used to predict the depth map at the corresponding scale. The final depth map is obtained by combining the predictions from all scales using a weighted combination. Similarly, the authors of the work presented in [8] proposed a method for estimating depth and surface normals from a single image. The network proposed in [8] includes a regression stage that uses a deep CNN model to learn mapping from multi-scale image patches to depth or surface normal values at the super-pixel level, which is obtained using the SLIC algorithm introduced in [9]. They converted the estimated super-pixel depth and surface normal to the pixel level by using potentials on the depth or surface normal maps, such as a data term, a smoothness term, and an auto-regression term characterizing the local structure of the estimated map. In turn, the authors of the work presented in [10] proposed a novel method for depth estimation from a single image. The method proposed in [10] uses a CNN to predict depth from an RGB image and then refines the depth predictions with an adaptive surface normal constraint. The normal surface constraint is computed by estimating the scene’s surface normals using the predicted depth map and comparing them to the surface normals estimated from the RGB image. The difference between these two estimates is then used to fine-tune the predicted depth map, yielding more accurate depth predictions.

In addition, the authors in [11] introduced an algorithm for estimating consistent dense depth maps using a CNN trained with geometric optimization for estimating smooth camera paths and precise and reliable depth reconstruction. In [12], the authors presented a DenseDepth network, a deep neural network that uses transfer learning to predict the depth value from the colour image directly. To create a high-resolution depth map, they used the pre-trained DenseNet backbone [13] along with bilinear up-sampling and skip connections on the decoder, while [2] developed a deep learning model consisting of two successive deep neural networks to estimate the depth of the main object presented in a single image. A dense depth map of a given colour image is estimated by the first network based on the generative neural network (GAN). The estimated depth map is then used to train a convolutional neural network (CNN) to predict the 3D pose of the object.

Recently, the authors of [14] suggested a brand-new component for a transformer-based depth estimation architecture called AdaBins. The depth range is divided into bins by the AdaBins block, and the centre value of each bin is adaptively estimated for each image. After this, linear combinations of the bin centres are used to estimate the final depth values. In [15], the authors presented a BinsFormer method to estimate depth from monocular images. Their model uses a transformer module to predict bins in a set-to-set manner, a per-pixel module to estimate high-resolution pixel-wise representations, and a depth estimation module to combine this information to predict final depth maps. The two methods, as mentioned above, achieved new state-of-the-art results, but it is computationally expensive, and the training settings for transformer-based models require many resources. Moreover, these models do not perform more generalisation than the other deep learning models of depth estimation.

All the methods mentioned above focus on simply extracting the image’s structure and content that cause blurring of the expected depth images. As a result, we can take advantage of the contextual semantic data that semantic segmentation models may gather. Therefore, we need to benefit from contextual semantic information that semantic segmentation models can extract. There are small trials for leveraging the semantic features to enhance depth estimation since information exchange between tasks has significant advantages, such as in [16]. The model suggested by [16] included a multi-scale skip connection with self-attentive modules to highlight the feature maps from the various objects during the decoding stage. In [17], the authors provided a useful framework for enhancing depth prediction accuracy when depth prediction and semantic labelling tasks are learned together. They created a feature-sharing module to combine discriminative features from various tasks, which helped the network comprehend the scene’s context and use correlated features to produce more precise predictions. To increase the accuracy of the results generated by a deep CNN, the authors of [18] trained a single network for both semantic and depth prediction. A fully connected conditional random field (CRF), which captures the contextual information, is coupled with the CNN to refine the estimated depth map. Additionally, many multi-task methods use semantic data to close the gap between the two tasks (i.e., depth estimation and semantic segmentation), e.g., [19,20,21]. These methods enhanced the depth features by sharing the content and context information between the two tasks. Consequently, this work attempts to present a deep learning network that can combine contextual and content information to predict more accurate depth estimation from a single image, maintaining object discontinuities and the details of multi-scale objects in the scene.

## 3. Methodology

As shown in Figure 2, the proposed model is based on two parallel networks—every network works as an autoencoder that can map between different domains. In particular, the first autoencoder network is learned to map from an RGB image to a depth image. The second one learns the multi-scale semantic features of the input image by classifying the image’s structural elements. We employ the HRNet-V2 network as the pre-trained model for the second autoencoder. The HRNET-V2 maintains high-resolution representations by connecting high-to-low-resolution convolutions in parallel and carrying out numerous multi-scale fusions across parallel convolutions. To reconstruct the original final depth map, a decoder network will be fed the concatenation of the features extracted by the two encoders. In order to optimize the network, the final estimated depth image is compared to a ground-truth depth image during the training stage using different loss functions illustrated in the following subsections.

### 3.1. Problem Formulation

Let a∈A be a 2D image. The problem of generating the corresponding depth image, b∈B, is formally defined as a function f:A→B that maps elements from the domain *A* to elements in the co-domain *B*. Our proposed model consists of three consequent networks, content encoder $E_{1}\left(A\right)$, semantic encoder $E_{2}\left(A\right)$, and decoder $D(\hat{A})$, where $\hat{A}$ is the combined features generated by EC and ES. The *B* is the final depth image of the last layer of the decoder, DE. In Equations (1)–(5), we explain the operation of the model’s workflow with the training and testing stages.

(1) $$F1=E_{1}\left(A\right),$$

where F1 is the features extracted from the $E_{1}$ encoder part in the autoencoder network, and *A* is the input image.

(2) $$F2=E_{2}\left(A\right),$$

where F2 is the contextual information extracted from the $E_{2}$ encoder part in the HRNet-v2 network, and *A* is the input image.

(3) F=F1⊕F2,

where *F* (or $\hat{A}$) is the concatenate of the features extracted in Equation (1) and the contextual information that has been extracted in Equation (2).

(4) R=D(F),

where *R* is the feature maps extracted from the *D* decoder part in the autoencoder network, and *F* is the concatenate of the features computed in Equation (3).

(5) Output=DE(R),

where Output is the final depth map extracted from the DE depth estimation layer in the network, and *R* is the feature maps extracted in Equation (4).

### 3.2. Network Architecture

The entire network comprises two networks, as shown in Figure 2: an autoencoder is used to extract structure and content features, and another is used to extract semantic features.

#### 3.2.1. Content Encoder

**Figure 3:**
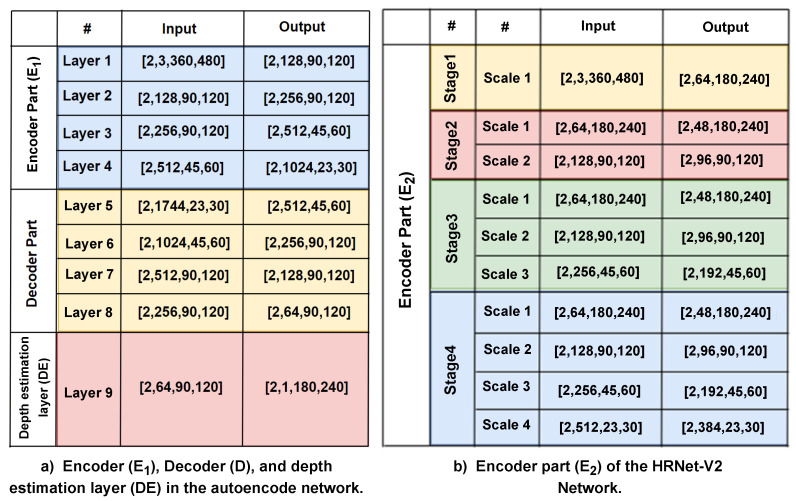
(**a**) Input and output sizes of each layer in the encoder $E_{1}$ and decoder *D* parts for the autoencoder network. (**b**) Input and output sizes of each scale in the encoder part $E_{2}$ of the HRNet-V2 Network. Colours correspond to the colours used in Figure 2.

An RGB image *a* is fed into the encoder $E_{1}$, which converts it into a state with a fixed shape that represents the features of the content and structure. The second component is a decoder that maps the encoded high-level features to a depth image. The input RGB image is encoded into a feature vector through the use of the SENet-154 [5], which was previously trained on ImageNet [22]. Our encoder consists of the first four blocks of the SENet, and we use the size of the input RGB images of 360×480 as shown in Figure 2. The first two layers downsample the original size of the input images to the quarter, producing 128 and 256 feature maps, respectively. The third block generates 512 feature maps with a size of 45×60. The final size of the high-level feature maps is 23×30×1024. To cope with overfitting, our model uses a dropout with a ratio of 0.2 and a label-smoothing regularisation proposed in [23] during the training stage. Likewise, to ensure consistency between training and testing, we froze the parameters of all the batch normalization (BN) layers. In Figure 3a, we show each layer’s input and output sizes for the network in the encoder layers.

#### 3.2.2. Semantic Encoder

For extracting the semantic features, we use the encoder $E_{2}$ as a pre-train model. The encoder network is based on a high-resolution representation network, “HRNet-V2”, a recently proposed model in [6] that can maintain high-resolution representations of multi-scale objects throughout feature extraction throughout the model without the traditional bottleneck design. The HRNet-V2 performs at the cutting edge on various pixel-labelling tasks. To achieve robust feature representations with minimal overhead, the HRNet-V2 model explores the representations from all high-to-low-resolution parallel convolutions as opposed to just the high-resolution representations. The HRNet-v2 network has four stages in total. There are high-resolution convolutions in the first stage. The second, third, and fourth stages are composed of repeating modularized multi-resolution blocks. A group of multi-resolution convolutions makes up a multi-resolution block. The convolution group, which divides the input channels into various groups of channels and conducts a regular convolution over each group over various spatial resolutions separately, is the foundation for the multi-resolution group convolution. It is comparable to the regular convolution’s multi-branch full-connection method. A regular convolution can be split into several smaller convolutions, as stated in [24]. Both the input and output channels are split up into a set of groups. Each connection between the input and output subsets is a complete convolution. Several 2-stride 3×3 convolutions are used in [25] to achieve the resolution reduction. Bilinear up-sampling is used in [25] to implement the resolution increase. We display the input and output sizes for each scale in the semantic encoder built on the HRNet-V2 network in Figure 3b.

#### 3.2.3. Decoder

The decoder *D* network comprises four deconvolution layers in total. Starting from the concatenation of the output of the content encoder and the output of the last layer from the encoder network of the semantic segmentation network, we perform a 1×1 deconvolution. Next, three 3×3 deconvolutions were added, with output filters set to have half the number of input filters. The feature maps are extended using an up-sampling block composed of a 2×2 bilinear up-sampling between the first three deconvolutions [26]. Except for the final layer, every layer of the decoder is followed by a leaky ReLU activation function with alpha=0.2 [27]. In turn, a ReLU activation follows the final layer block. The output of the previous layer of the decoder with the output of the encoder’s corresponding layers for a skip connection and a coarser depth map produced by the depth estimator layer are concatenated as the input to the next deconvolution. The final layer is a depth estimator for the finest depth map DE with a size of 240×180×1. Figure 3 shows the input and output sizes for the network’s decoder layers.

### 3.3. Loss Functions

Similar to [12], we formulate our monocular depth estimation problem as the minimization of a re-projection error between the estimated depth $\hat{B}\left(x,y\right)$ and the ground-truth B(x,y) at the time of training. Our objective loss function composes of three loss functions.

In our model, the main objective for combining these three loss functions into a single objective loss function is to combine the benefits of each loss function to improve the model’s overall performance. The $L_{1}$ loss measures the absolute difference between the predicted and ground-truth values, which is robust to outliers but lacks sensitivity to perceptual similarity. On the other hand, The SSIM loss measures the structural similarity between the predicted and ground-truth images, which is sensitive to perceptual similarity but less robust to outliers. In turn, the MSE loss measures the mean-squared difference between the predicted and ground-truth values, which is commonly used but can be sensitive to outliers. By combining these loss functions, the model can take advantage of their individual strengths and overcome their weaknesses, resulting in better accuracy and robustness. Each loss function can capture different aspects of the problem being solved, such as accuracy, robustness, or generalization. Additionally, the three losses are frequently used in state-of-the-art depth estimation. The three loss functions can be defined as follows:

The point-wise L1-norm defined by the depth values is the first content loss $L_{L1}$ that can be defined as follows:

(6) $$L_{L1}\left(B,\hat{B}\right)=\frac{1}{wh}(\sum_{x=1}^{w}\sum_{y=1}^{h}|B\left(x,y\right)-\hat{B}\left(x,y\right)\left|\right),$$

where *w* and *h* are the width and height of the ground-truth depth, respectively.

The expected perceptual quality of the digital images is assessed using the structural similarity index measure (*SSIM*) loss index. The *SSIM* loss function is a complete reference metric used to assess the accuracy of the depth images generated compared to the corresponding ground-truth values. The *SSIM* index $L_{SSIM}$ can be defined as:

(7) $$L_{SSIM}\left(B,\hat{B}\right)=\frac{1}{2}\left(1-\frac{\left(2\mu_{\hat{B}}\mu_{B}+c_{1}\right)\left(2\sigma_{\hat{B}B}+c_{2}\right)}{\left(\mu_{\hat{B}}^{2}+\mu_{B}^{2}+c_{1}\right)\left(\sigma_{\hat{B}}^{2}+\sigma_{B}^{2}+c_{2}\right)}\right),$$

where $\mu_{\hat{B}}$ is the mean of $\hat{B}$, $\sigma_{\hat{B}}$ is the standard deviations of $\hat{B}$, $\mu_{B}$ is the mean of *B*, $\sigma_{\mu_{B}}$ is the standard deviations of *B*, $\sigma_{\hat{B}B}$ is the covariance of $\hat{B}$, and $c1=0.01^{2}$ and $c2=0.03^{2}$.

The mean-square error (*MSE*) is the third loss function ($L_{MSE}$) can be defined as:

(8) $$L_{MSE}\left(B,\hat{B}\right)=\frac{1}{wh}\left(\sum_{x=1}^{w}\sum_{y=1}^{h}{\left(B\left(x,y\right)-\hat{B}\left(x,y\right)\right)}^{2}\right).$$

Our final objective function used for training the proposed model, $L(B,\hat{B})$, including the three mentioned loss functions, can be defined as follows:

(9) $$\begin{array}{c} \begin{array}{c} L\left(B,\hat{B}\right)=\alpha L_{L1}\left(B,\hat{B}\right)+\beta L_{SSIM}\left(B,\hat{B}\right)+\gamma L_{MSE}\left(B,\hat{B}\right), \end{array} \end{array}$$

where α, β and γ are weighting factors empirically set to 0.2, 0.5 and 0.3, respectively.

## 4. Experiments and Results

This section outlines the experiments conducted to assess the developed model and evaluation metrics applied to quantify the model’s performance.

### 4.1. Dataset

The NYU Depth-v2 [28] and SUN RGB-D [29] datasets are two publicly available indoor datasets used for testing state-of-the-art depth estimation from monocular images and evaluating the performance of our model. To train the developed network, we used the NYU Depth-v2 dataset. We evaluated the trained model without further fine-tuning using the SUN RGB-D dataset to assess the model generalization.

#### 4.1.1. NYU Depth-v2 Dataset

The performance of the proposed model has been thoroughly tested in this work using the publicly available NYU Depth-v2 dataset, which contains images and depth maps for various indoor scenes captured at a resolution of 640×480 [28]. The ground-truth depth maps have a maximum resolution of 10 metres. The dataset includes 654 testing samples and 120,000 raw frames for training. We use this dataset to train our model on a portion of photorealistic indoor scenes with a training set of 50,000 and a testing set of 654, along with the corresponding ground-truth depth maps, as suggested in [12]. All images are reduced in size from 640×480 to 480×360 before being fed into the deep model.

#### 4.1.2. SUN RGB-D Dataset

The public SUN RGB-D dataset is used in this study to provide RGB images, and depth maps for various indoor scenes with a resolution of 730×530, with depth maps having a maximum resolution of 10 m. This dataset is used to test the generalizability of the model. The dataset includes 5050 testing samples and 10K images with a high scene diversity collected with four sensors for training. We do not train the proposed model using this dataset; it is only used for evaluation and validation. Without fine-tuning or additional adjustments, we cross-evaluate the trained model by the NYU dataset on the test set of 5050 images. All images are reduced in size from 730×530 to 480×360 before inputting into the network.

### 4.2. Parameter Settings

We used the ADAM optimizer introduced in [30] to train our model with parameters of $beta_{1}=0.5$, $beta_{2}=0.999$, and an initial learning rate of 0.0001. The optimal combination was with a batch size of 2 and 15 epochs. The PyTorch [31] deep learning framework was used to run all experiments on a 64-bit Core i7-6700, 3.40 GHz CPU with 16 GB of memory, and an NVIDIA GTX 1080 GPU under Ubuntu 16.04. The proposed model’s computational cost for the training process is about 2.5 h per epoch with a 2 batch size. The performance of the online depth map estimation is around 0.028 s.

### 4.3. Evaluation Measures

We assessed the performance of the proposed model by estimating errors below a pre-determined threshold between the estimated depth map and the ground-truth to demonstrate how frequently our prediction is accurate. For instance, we used a threshold accuracy proposed in [32] assuming that a given error will be less than a threshold $thr^{Z}$. The threshold accuracy can be defined as:

(10) $$\delta_{Z}=E_{T}\left[\;F\left(\max\left(\frac{B_{\left(i\right)}}{{\hat{B}}_{\left(i\right)}},\frac{{\hat{B}}_{\left(i\right)}}{B_{\left(i\right)}}\right)<thr^{Z}\right)\right]\;$$

where F(·) is an indicator function that returns either 0 or 1. We set thr=1.25, and Z∈{1,2,3} similar to [32].

As part of our quantitative assessment, we report any errors calculated using three popular metrics. The root-mean-square (*RMS*) error, which provides a quantitative measure of the per-pixel error, is the first measurement, and the average relative (*Rel*) error is the second. The average $log_{10}$ error is the third metric employed to assess the overall performance. The three measures as mentioned earlier are best described as follows:

(11) $$RMS=\sqrt{\frac{1}{n}\sum_{i=1}^{n}{\left(B_{\left(i\right)}-{\hat{B}}_{\left(i\right)}\right)}^{2}},$$

(12) $$Rel=\frac{1}{n}\sum_{i=1}^{n}\frac{|B_{\left(i\right)}-{\hat{B}}_{\left(i\right)}|}{B_{gt(i)}},$$

(13) $$log_{10}=\frac{1}{n}\sum_{i=1}^{n}\left|log_{10}\left(B_{\left(i\right)}\right)-log_{10}\left({\hat{B}}_{\left(i\right)}\right)\right|,$$

### 4.4. Results and Discussion

#### 4.4.1. Ablation Study

First of all, we performed an ablation study on our proposed model on the NYU Depth-v2 dataset under various measures to demonstrate the effects of different improvements in the baseline autoencoder model:

1. Baseline that has one autoencoder network as proposed in [12] with the point-wise L1-norm and SSIM losses.
2. Baseline with skip connection: Applying skip connection to the autoencoder network by feeding the features maps extracted by the encoder layers to the corresponding decoder layers.
3. Proposed model: The baseline with skip connection and the feature extracted by the encoder of the semantic segmentation autoencoder.

**Figure 4:**
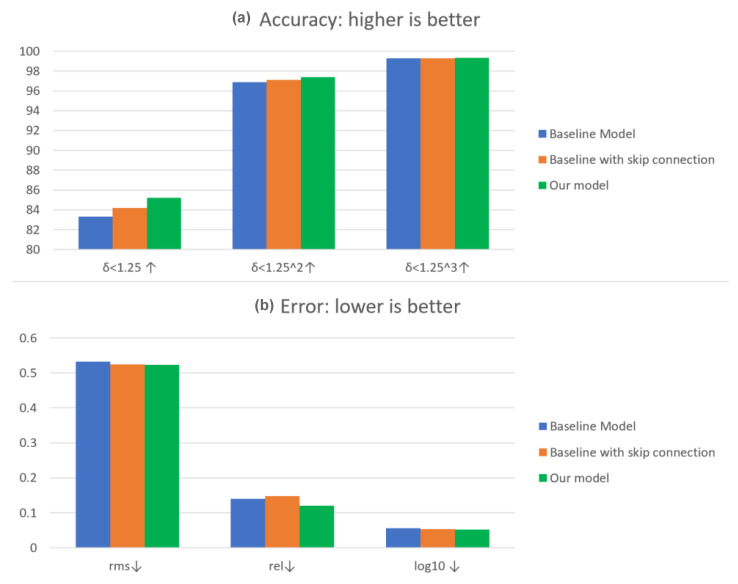
(**a**) The accuracy and (**b**) the three error measures of the three variations of our model with the NYU Depth-v2 dataset (green); baseline (blue), and baseline with skip connection (orange).

In Table 1, the quantitative results with the NYU Depth-v2 dataset are shown. The proposed model’s performance yielded better results than its variations in terms of accuracy of $\delta_{Z}$, RMS, Rel and $log_{10}$ errors. Furthermore, the accuracy $\delta_{Z1.25}$ improved by 1.03%, and Rel error improved by 0.02% compared to the second-best results of the baseline with the skip connection model. Compared to the baseline method, merging the semantic features with the content features yielded a significant improvement with $\delta_{Z}$ of 2%. Furthermore, in Figure 4, we give examples of estimated depth obtained from the NYU Depth-v2 testing set. More precisely, the accuracy and error percentage between our model and the rest of the models in the ablation study.

For evaluating the generalization of the proposed model, in Table 2 we show the quantitative results of the ablation study with the SUN RGB-D dataset. The proposed model’s performance yielded better results than its variations in terms of accuracy of $\delta_{Z}$, RMS, Rel and $log_{10}$ errors. The accuracy $\delta_{Z1.25}$ improved by 1.1%, and Rel error improved by 0.05% compared to the second-best results of the baseline with the skip connection model. Compared to the baseline method, merging the semantic features with the content features yields a significant improvement with $\delta_{Z}$ of 1.7%. Thus, merging the content features with the contextual features yielded more accurate depth estimation.

**Figure 5:**
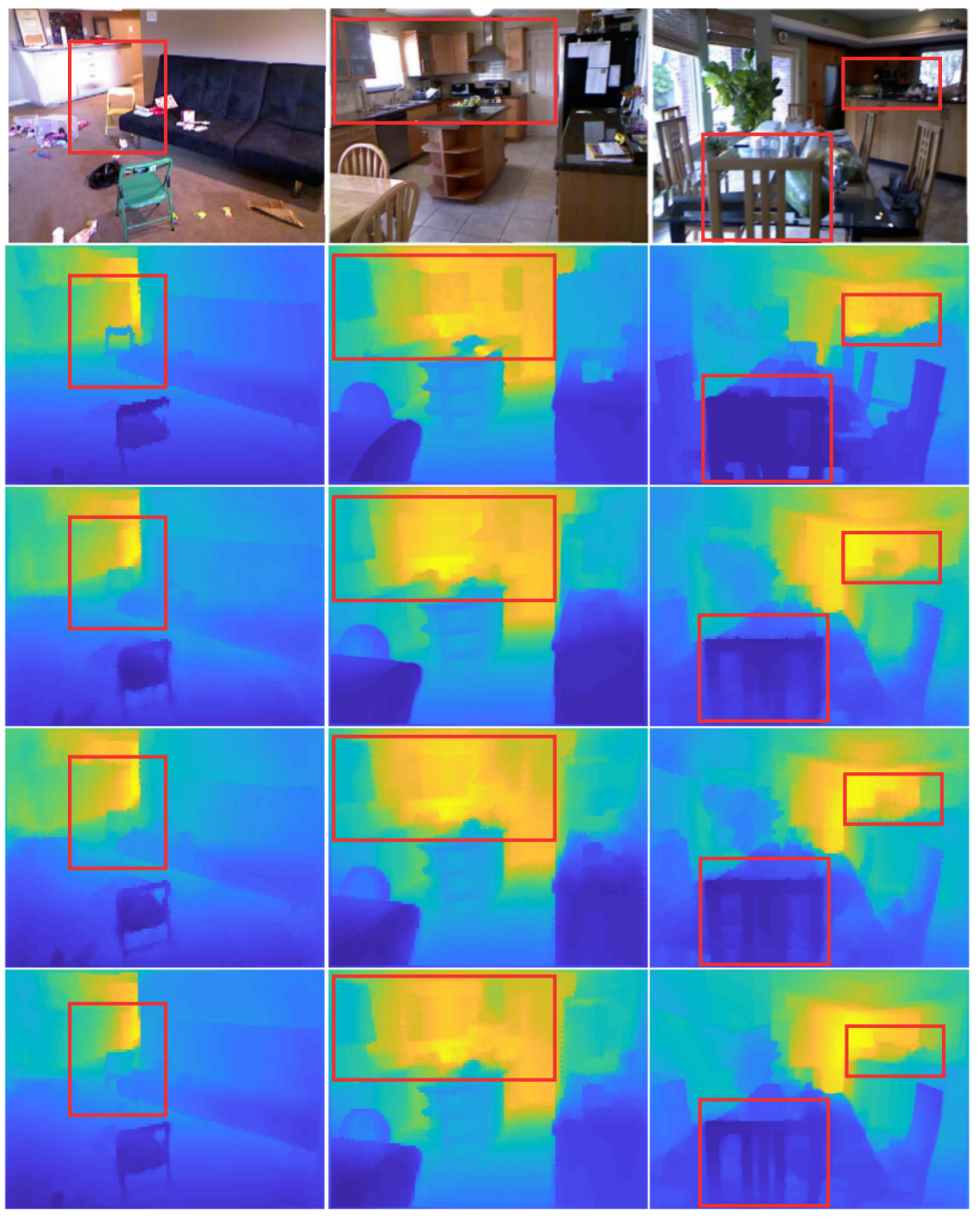
Examples from the test NYU Depth-v2 dataset of depth estimates with baseline, baseline with skip connection and our model. For each image, we show (row 1) the input image, (row 2) the ground-truth, (row 3) the output for the baseline model, (row 4) the output for the baseline with skip connection, and (row 5) the final estimate depth image with our model.

To more thoroughly assess the proposed model’s effectiveness, we randomly selected images from the NYU Depth-v2 test set to demonstrate the proposed model’s ability to estimate accurate depth maps (see Figure 5). It is worth noting that our model can generate depth maps that include details that the baseline models do not include. By integrating two autoencoders for depth estimation and semantic segmentation, the model learned the correct cardinality (i.e., objects) inside the images. Our model can generally estimate correct depth values for small objects presented in the scene (see Figure 5, Column 1) and far away from the camera (see Figure 5, Column 2). It can also properly detect the discontinuities of the objects, even for objects whose colours are similar to those of the background (see Figure 5, Column 3).

In general, our model can estimate correct depth values for objects that are small (see Column 1) and for objects that are far away from the camera (see Column 2), as well as detect the boundaries between objects whose colours are similar to the background (see Column 3).

**Figure 6:**
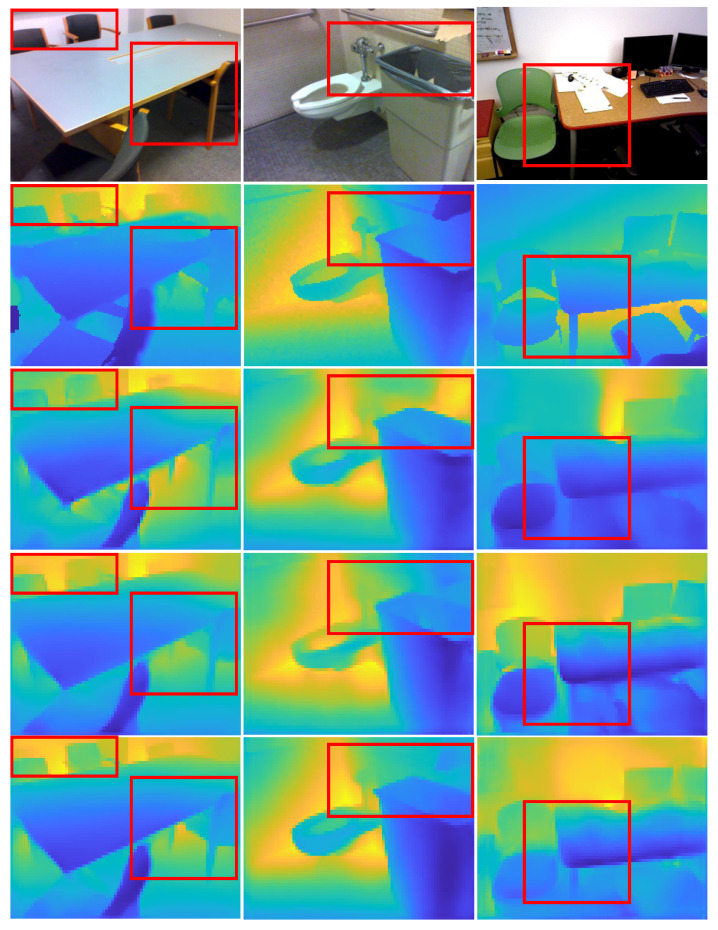
Examples from the test SUN RGB-D dataset of depth estimate with baseline, baseline with skip connection and our model. For each image, we show (row 1) the input image, (row 2) the ground-truth, (row 3) the output for the baseline model, (row 4) the output for the baseline with skip connection, and (row 5) the final estimated depth image with our model.

To generalize the proposed model’s performance on a concrete case, we tested it with the SUN RGB-D dataset without fine-tuning. We randomly selected some images from the dataset to demonstrate the proposed model’s ability to estimate depth maps and compare the results to the baseline and baseline with skip connection models. (see Figure 6). Again, our proposed model can preserve the discontinuities of the objects, even for small objects.

#### 4.4.2. Analysing Performance

Secondly, we compared the proposed model with four state-of-the-art methods [12,33,34,35]. We show evaluation measures on the NYU Depth-v2 dataset with the four tested approaches and the proposed model in Table 3. The proposed model outperformed the four methods in terms of the three measures ($\delta_{Z}$ of a threshold of 1.25, $1.25^{2}$ and $1.25^{2}$, and Rel and the $log_{10}$ error). $\delta_{Z}$ of a threshold of 1.25 with our model was improved by 0.72% compared to [34], the second-best method. In turn, with $\delta_{Z}$ of $1.25^{2}$, [12], our method achieved an improvement of 0.7% compared to the other three methods. Furthermore, our model reduces the Rel error by 0.02% compared to [12], the second-best method. Additionally, the proposed method improves the $log_{10}$ error by 0.004% compared to [12], the second-best method. The model proposed in [12] yielded the best accuracy for the RMS error, higher than our proposed model with a difference of 0.057%.

Table 4 shows the evaluation measures with the SUN RGB-D dataset with the proposed model and five state-of-the-art monocular depth estimation models [14,15,36,37,38]. The significant improvement in most of the metrics in Table 4 indicates an outstanding generalization of the proposed model. The proposed model was superior in terms of $delta_{Z}\left(thr=1.25\right)$, Rel, RMS, and $log_{10}$. $delta_{Z}\left(thr=1.25\right)$ showed an improvement of 3.2% compared to second-best model [15]. Ref [15] yielded an improvement in $delta_{Z}\left(thr=1.25^{2}\right)$ and $delta_{Z}\left(thr=1.25^{3}\right)$ of 1.3% and 1.6%, respectively, compared to our model. Furthermore, with the Rel error, our proposed model yielded an improvement of 0.007% compared to the second-best method [15]. In turn, the model presented in [15] yielded the lowest error rates of RMS and $log_{10}$, which is a bit lower than our proposed model with differences of 0.001%, and 0.029%, respectively. However, our method provided the best accuracy in most measures compared to the second-best model. Notice that the second-best model is trained on an input image size more significant than our model, with a batch size of 16, compared to our model with 2 batch sizes only.

Finally, we demonstrate some of the outcomes from the SUN RGB-D dataset in Table 4. More specifically, the results show how our model can deliver outcomes comparable to those of cutting-edge models. Our model provided the best $delta_{Z}\left(thr=1.25\right)$ and the lowest Rel rate among the eight methods. In turn, the BinFormer model proposed in [15] provided the best results with $delta_{Z}\left(thr=1.25^{2}\right)$, $delta_{Z}\left(thr=1.25^{3}\right)$, RMS and $log_{10}$. It is worth saying that $delta_{Z}\left(thr=1.25\right)$ is a more restricted measure than $delta_{Z}\left(thr=1.25^{2}\right)$ and $delta_{Z}\left(thr=1.25^{3}\right)$. The BinFormer model also depends on different transformers modules that are more complex than the CNNs. Furthermore, in contrast to our model’s standard loss functions, the BinFormer relies on the SILog error metric introduced by [7] to measure the relationship between points in the scene regardless of the absolute global scale, helping detect accurate depth maps.

**Figure 7:**
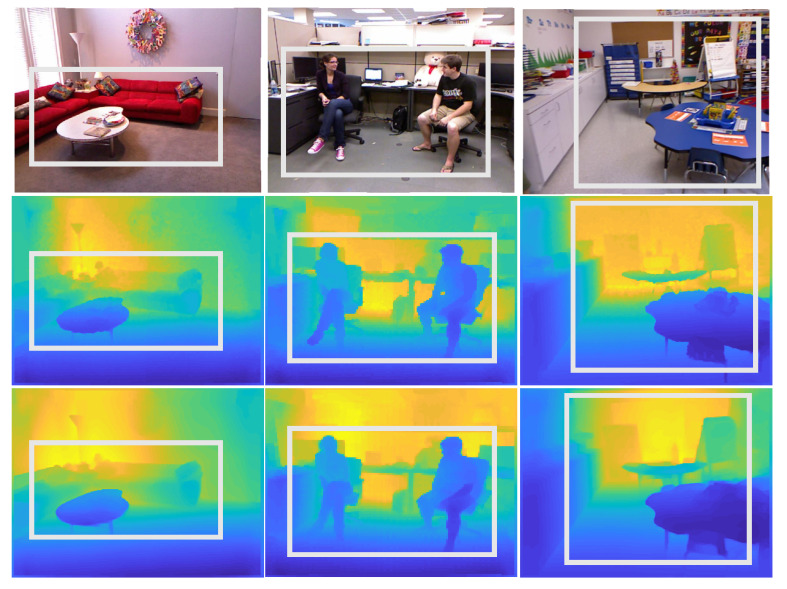
(row 1) Input images, (row 2) ground-truth depth, and (row 3) resulting depth images with the NYU Depth-v2 dataset.

**Figure 8:**
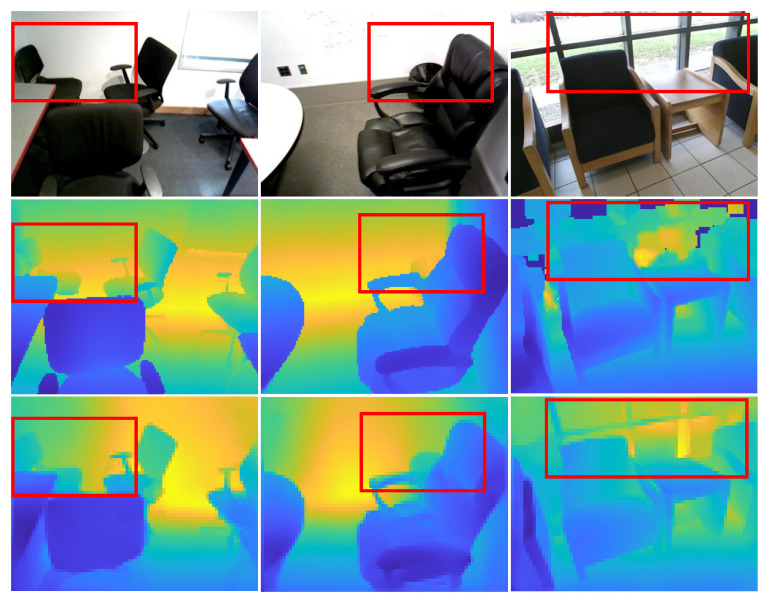
(row 1) Input images, (row 2) ground-truth depth, and (row 3) resulting depth images with the SUN RGB-D dataset.

In Figure 7 and Figure 8, with the NYU Depth-v2 and SUN RGB-D datasets, we show examples of input, ground-truth depth, and generated depth images. As demonstrated, our model can predict a depth image very close to the reference ones while preserving the objects’ discontinuities and small details. Our model keeps the outline of the objects in the scenes so that they can be recognized directly from the depth maps. In contrast, object outlines appear crumbled in the depth maps generated by other tested techniques.

## 5. Conclusions and Future Directions

This paper proposes a deep model for predicting precise depth maps from monocular images by integrating two autoencoders to extract the content and contextual information. The model combines the features extracted by the content encoder with those extracted by the second semantic segmentation encoder and feeds them into a decoder network to reconstruct the depth images. The model’s performance was evaluated on two publicly available datasets, SUN RGB-D and NYU Depth v2, yielding promising results for predicting depth images from monocular images with high precision and an acceptable computational cost. Our proposed approach significantly outperformed several state-of-the-art monocular depth estimation techniques, achieving an accuracy of 85% while minimizing three errors of Rel by 0.12, RMS by 0.523, and $log_{10}$ by 0.0527. In the future, we plan to expand on this work by exploring how our proposed model can be used for pose estimation and volume calculation using a monocular vision system. These tasks are essential in many applications, such as robotics, where accurately estimating an object’s orientation and size is critical. By leveraging the power of deep learning and semantic segmentation, our proposed model has the potential to achieve outstanding results in these areas. Moreover, we plan to investigate how our proposed approach can be further optimized to improve its performance, reduce computational costs, and be generalize to different datasets and environments. We also plan to explore the possibility of incorporating other data modalities, such as LiDAR or RGB-D cameras, to further enhance our depth estimation model’s accuracy.

## Figures and Tables

**Table 1:** Quantitative results of the ablation study on the NYU Depth-v2 dataset.

Method	Accuracy: Higher Is Better			Lower Is Better		
	δ<1.25 ↑	$\delta <1.25^{2}$ ↑	$\delta <1.25^{3}$ ↑	*Rel*↓	*RMS* ↓	$\log_{10}$ ↓
Baseline Model	0.833	0.969	0.9928	0.14	0.532	0.056
Baseline with skip connection Model	0.842	0.971	0.9931	0.148	0.525	0.054
Our model	**0.8523**	**0.974**	**0.9935**	**0.121**	**0.523**	**0.0527**

**Table 2:** Quantitative results of the ablation study on the SUN RGB-D dataset without fine-tuning.

Method	Accuracy: Higher Is Better			Lower Is Better		
	δ<1.25 ↑	$\delta <1.25^{2}$ ↑	$\delta <1.25^{3}$ ↑	*Rel* ↓	*RMS* ↓	$\log_{10}$ ↓
Baseline Model	0.82	0.945	0.972	0.144	0.46	0.066
Baseline with skip connection Model	0.826	0.948	0.973	0.141	0.46	0.064
Our model	**0.837**	**0.950**	**0.974**	**0.136**	**0.45**	**0.062**

**Table 3:** Quantitative results of the proposed model and four depth estimation methods on the NYU Depth v2 dataset.

Method	Accuracy: Higher Is Better			Lower Is Better		
	δ<1.25 ↑	$\delta <1.25^{2}$ ↑	$\delta <1.25^{3}$ ↑	*Rel* ↓	*RMS* ↓	$\log_{10}$ ↓
Hao et al. [33]	0.841	0.966	0.991	0.127	0.555	0.053
Ramamonjisoa et al. [34]	0.8451	0.9681	0.9917	0.1258	0.551	0.054
Alhashim et al. [12]	0.846	0.97	0.99	0.123	**0.465**	0.053
Tang et al. [35]	0.826	0.963	0.992	0.132	0.579	0.056
Our model	**0.8523**	**0.974**	**0.9935**	**0.121**	0.523	**0.0527**

**Table 4:** Results of the model trained on the NYU-Depth-v2 dataset and tested on the SUN RGB-D dataset [29] without fine-tuning.

Method	Encoder	Accuracy: Higher Is Better			Lower Is Better		
		δ<1.25 ↑	$\delta <1.25^{2}$ ↑	$\delta <1.25^{3}$ ↑	*Rel* ↓	*RMS* ↓	$\log_{10}$ ↓
Chen et al. [36]	SENet-154	0.757	0.943	0.984	0.166	0.494	0.071
Yin et al. [37]	ResNeXt-101	0.696	0.912	0.973	0.183	0.541	0.082
BTS. [38]	DenseNet-161	0.740	0.933	0.980	0.172	0.515	0.075
Adabins. [14]	E-B5+Mini-ViT	0.771	0.944	0.983	0.159	0.476	0.068
BinsFormer. [15]	ResNet-18	0.738	0.935	0.982	0.175	0.504	0.074
BinsFormer. [15]	Swin-Tiny	0.760	0.945	0.985	0.162	0.478	0.069
BinsFormer. [15]	Swin-Large	0.805	**0.963**	**0.990**	0.143	**0.421**	**0.061**
Our model	SENet-154	**0.837**	0.950	0.974	**0.136**	0.45	0.062

## Data Availability

Not applicable.

## Abbreviations

The following abbreviations are used in this manuscript:

Abs Rel	Absolute and Relative Error
ADAM	Adaptive Moment Estimation Optimization Method
BL	Base Line
BLSC	Base Line withSkip Connections
BN	Bbatch Nnormalization
DE	Depth Estimation
E1	Content Encoder
E2	Semantic Encoder
MDE	Monocular Depth Estimation
MedErr	Median Error
MLF	Multi Loss Function
MRF	Markov Random Field
MSE	Mean Squared Error
Rel	Relative Error
ReLU	Rectified Linear Unit
RMSE	Root Mean Square Error
SCLoss	Semantic Context Loss
SCs	Skip Connections
SENets	Squeeze-and-Excitation Networks
SSIM	Structural Similarity
